# Putative HIV and SIV G-Quadruplex Sequences in Coding and Noncoding Regions Can Form G-Quadruplexes

**DOI:** 10.1155/2017/6513720

**Published:** 2017-12-31

**Authors:** Petra Krafčíková, Erika Demkovičová, Andrea Halaganová, Viktor Víglaský

**Affiliations:** Department of Biochemistry, Institute of Chemistry, Faculty of Sciences, P. J. Safarik University, 04001 Kosice, Slovakia

## Abstract

The HIV virus is one of the most studied viruses in the world. This is especially true in terms of gene sequencing, and to date more than 9 thousand genomic sequences of HIV isolates have been sequenced and analyzed. In this study, a series of DNA sequences, which have the potential to form G-quadruplex structures, is analyzed. Several such sequences were found in various coding and noncoding virus domains, including the U3 LTR, tat, rev, env, and vpx regions. Interestingly, a homological sequence to the already well-known HIV integrase aptamer was identified in the minus-strand. The sequences derived from original isolates were analyzed using standard spectral and electrophoretic methods. In addition, a recently developed methodology is applied which uses induced circular dichroism spectral profiles of G-quadruplex-ligand (Thiazole Orange) complexes to determine if G-rich sequences can adopt G-quadruplex structure. Targeting the G-quadruplexes or peptide domains corresponding to the G-rich coding sequence in HIV offers researchers attractive therapeutic targets which would be of particular use in the development of novel antiviral therapies. The analysis of G-rich regions can provide researchers with a path to find specific targets which could be of interest for specific types of virus.

## 1. Introduction

The human immunodeficiency virus (HIV) is an RNA retrovirus in the Retroviridae family which causes HIV infection and over time can lead to acquired immunodeficiency syndrome (AIDS). The virus belongs to the single-stranded positive-sense RNA* Lentivirus* genus and is formed from two molecules of genomic RNA that are converted into double-stranded DNA by the viral reverse transcriptase. The resulting viral DNA is then inserted into the cellular DNA by the HIV integrase. Once integrated, transcription from the proviral promoter at the 5′ long terminal repeat (LTR) generates mRNAs which code viral proteins and genomic RNA. The integrated provirus may become latent and the infected host cell can remain undetected by the immune system. To date, more than nine thousand HIV-1, SIV, and HIV-2 subtypes have been completely or partially sequenced. The genomic HIV contains regions rich in G-residues which show a marked tendency to adopt G-quadruplex structures, and a number of studies published in recent years have revealed the biological significance of G-quadruplexes in HIVs [1–7]. G-quadruplexes have long been seen as a highly promising target for the development of new anticancer therapies [8, 9], but recent work has also suggested the possibility of adopting a similar strategy for the development of antiviral therapies. Formation of G-quadruplex is usually linked to moderate DNA transcription [10, 11]. Research devoted to G-quadruplexes has so far been limited to the field of viral genomes, despite the advantages of their small size and often naturally occurring double-stranded circular (episomal) form. One important study found that the G-quadruplex Epstein−Barr virus could disrupt the interaction of EBV nuclear antigen 1 with RNA. The linking regions of EBNA1 LR1 and LR2 were revealed to have a strong preference for G-quadruplex RNA and it was revealed that G-quadruplex RNA-interacting drugs block the functions of EBNA1 that are critical for viral DNA replication and episome maintenance [12, 13].

Recently it was confirmed that consensus sequences forming stable G-quadruplex structures are responsible for RNA replication and inhibition of protein translation of hepatitis C virus [14].

Our recently published results have also highlighted the significance of some G-rich regions in regulating areas with the ability to form stable G-quadruplexes in papilloma viruses [15].

G-quadruplex structures also seem to be critical for HIV-1 infectivity and could represent novel targets for antiviral drug development. For example, it is known that mutations disrupting G-quadruplex formation can enhance HIV promoter activity in cells and that treatment with G-quadruplex ligands decreases promoter activity and displays antiviral effects [6]. The U3 region contains a G-rich sequence ~80 nucleotides upstream from the transcription-starting site (TSS) and close to the TATA box. This sequence overlaps three SP1 binding sites which play a crucial role in the initiation of transcription [1, 4]. Recent research has confirmed the interaction between the Sp1 protein and a fragment of the HIV-1 promoter sequence folded into a G4 [16], and the effect of point mutations which disrupt the G-quadruplexes formed in the promoter has been analyzed [6].

Retroviral RNAs are now known to dimerize via G-rich regions in the cytoplasm of infected cells allowing two copies of the genome which is encapsidated in the newly produced virion [17]. The mechanisms which drive RNA dimerization play a role during the strand transfers which may be partially responsible for the viral variability through the production of recombinant molecules [18, 19].

G-rich sequences can form bimolecular G-quadruplex structures in the gag region of the HIV-1 genome, in close proximity to the dimer initiation site (DIS) [16, 20–22]. It has recently been shown that the recombination in the U3 domain is cation-dependent and is significantly lower in the presence of lithium ions, ions which are known to destabilize G-quadruplexes [23].

G-quadruplexes derived from the sequence of the negative regulatory factor (Nef) of HIV-1 were recently analyzed in vitro [5]. Nef G-quadruplexes repress the Nef expression and this finally results in a decrease in viral replication. Thus, targeting the G-quadruplexes located in the Nef coding sequence could lead to further attractive therapeutic opportunities.

**Figure 1:**
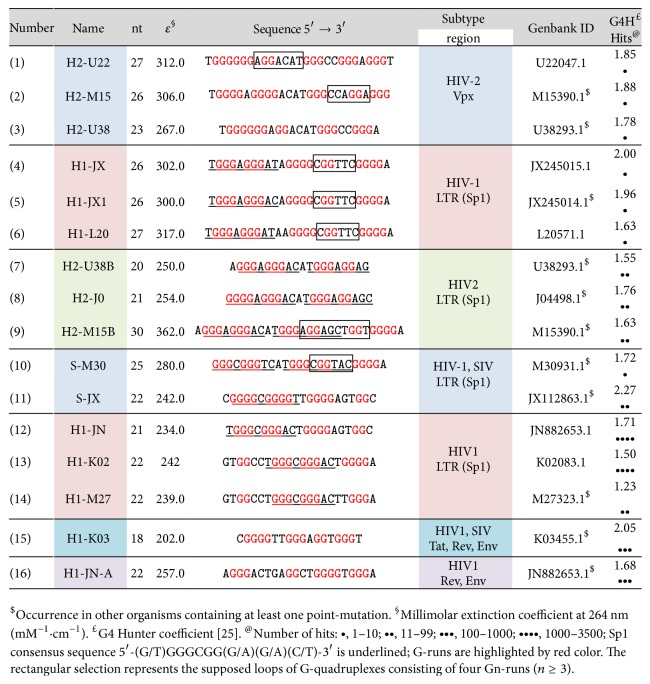
DNA oligonucleotides used in this study originating from HIVs and SIVs.

Therefore, the main goal of this study is to scrutinize HIV provirus genomes in an attempt to find G-rich regions which may be prone to forming G-quadruplex motifs. Several tools and strategies are available to predict G-quadruplex propensity from some sequences, but there are disadvantages and limitations associated with each algorithm [24–27]. Within the last ten years, it has become generally accepted that stable G-quadruplexes are mainly formed in G-rich regions consisting of four G-runs that contain two or more continuous guanosine residues (G_2–4_) interrupted by 1–7 nucleotides (G_2–4_N_*i*_G_2–4_N_*j*_G_2–4_N_*k*_G_2–4_) [28, 29]. The inner core of G-quadruplexes is based on the stacking of two or more G-tetrads, although some sequences, such as the VEGF aptamer, do not form in this way; this aptamer contains a loop which lacks any nucleotide residue [30]. Initially, our study was aimed at focusing on putative sequences (G_3_N_*i*_G_3_N_*j*_G_3_N_*k*_G_3_) which are able to form G-quadruplexes consisting of three G-quartets connected with loops shorter than 5 residues, but no sequences matching this criterion were found in the various HIV/SIV proviral genomic sequences which we prescreened randomly. Therefore, a different condition for finding G-quadruplex forming sequences in HIV genomes was applied: the desired sequences must consist of three G-runs (G_*n*_, *n* ≥ 3) and one dinucleotide island GG; see more details in Material and Methods. In fact, the existence of stable G-quadruplexes containing only 3 continuous G-runs has recently been confirmed, and this sequential motif can form so-called bulged G-quadruplexes [31]. Many such sequences have been found in various regions of HIV genomes, and those which are analyzed in this study are summarized in Figure 1. The formation and structure of G-quadruplexes of each oligonucleotide were verified using UV and CD spectroscopy and electrophoretic separation in the presence of either sodium or potassium ions. In order to exclude the false confirmation of G-quadruplex formation on the base of the CD spectra profiles alone, CD melting curves were also determined because the stability of all known G-quadruplexes is significantly higher in the presence of potassium than in the presence of sodium ions.

## 2. Material and Methods

All chemicals and reagents were obtained from commercial sources. DNA oligomers were obtained from Metabion, Germany (Figure 1). PAGE purified DNA was dissolved in double distilled water prior to use. Thiazole Orange was purchased form Sigma-Aldrich (cat. number 390062). Single-strand concentrations were determined precisely by measuring absorbance (~260 nm) at 95°C using molar extinction coefficients [15]. DNA concentration was determined using UV measurements carried out on a Jasco J-810 spectropolarimeter (Easton, MD, USA). Cells with optical path lengths of 10 mm were used, and the temperature of the cell holder was controlled with an external circulating water bath.

### 2.1. G-Quadruplex Searching Criteria

**Figure 2:**
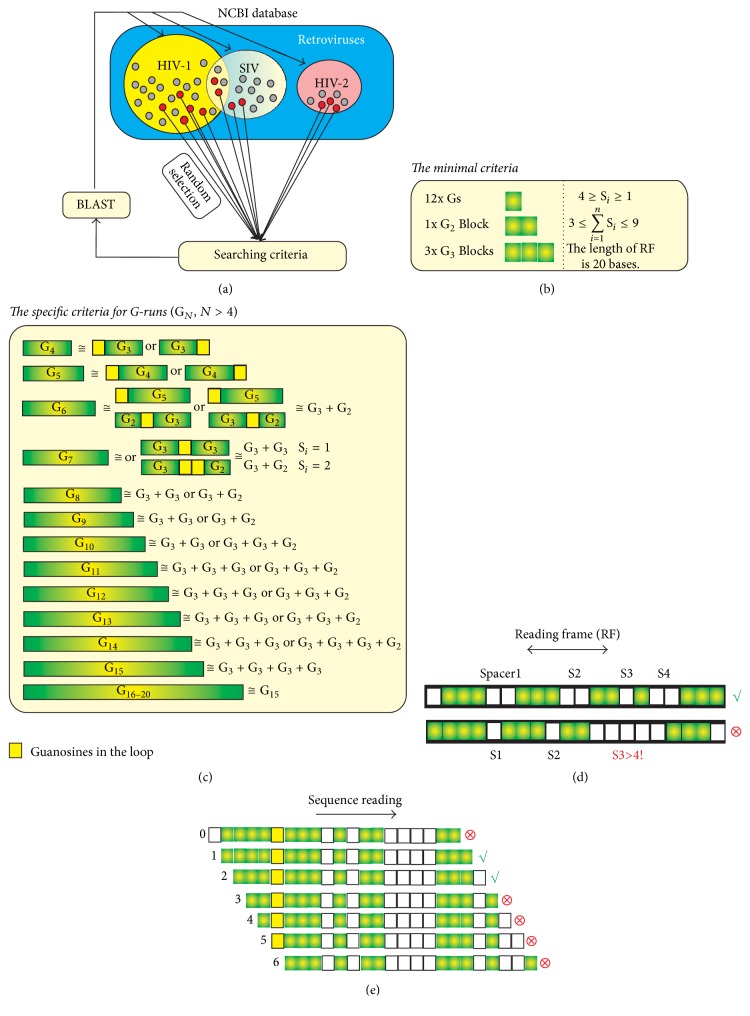
Strategy and searching criteria of putative G-quadruplex sequences. Randomly selected genomic sequences of HIV-1, HIV-2, and SIV from NCBI Gene database were analyzed (panel (a)). The criteria used to determine putative G-quadruplex sequences are listed in panel (b). ∑*S*_*i*_ represents the total number of nucleotide residues between G-runs (G_3_ and G_2_), and this value is restricted at the interval 〈3–9〉. Specific criteria were applied in cases in which G-runs consisted of more than four Gs (c); for instance, G_7_ is formed of either two G_3_ islands interrupted with one G (yellow square) or one G3 and G2 connected with two “yellow” Gs. The occurrence of putative sequences was researched by BLAST in other viruses listed in the Gene database. Positive and negative examples of the search procedure are shown in panels (d) and (e).

The search criteria for G-quadruplex forming sequences were restricted to sequences which possessed three continuous G-runs containing at least three neighboring Gs and one G-run containing only two neighboring Gs. We aimed to identify sequences with 1–4 nucleotides occurring between two continuous G-runs and with fewer than 9 nucleobases between G-runs in total; thus, the total required number of Gs was set at a minimum of 12. Initially, the reading frame of DNA was adjusted to 20 nucleotides (Figure 2).

The sequences fulfilling these criteria were considered as putative G-quadruplex forming sequences. If an additional G-run was located in close proximity (i.e., less than 3 nucleotides) to the putative sequence, it was also judged to be suitable for inclusion. In principle, we applied criteria similar to those utilized by QGRS mapper and the more comprehensive mining tool QuadBase2, a program which predicts G-quadruplex forming G-rich sequences (QGRS) in nucleotide sequences [27, 32]. The scores of G-quadruplex putative sequences found in HIV genomes were also analyzed using G4Hunter strategy (Figure 1) [25]. It is important to note that QGRS mapper and G4Hunter strategies can miss many sequences which were found by our access.

The randomly selected complete genomic sequences of 20 different HIV-1, 5 HIV-2, and 5 SIV viruses were analyzed, thereby identifying the sequences listed in Figure 1. The name of the oligonucleotide was derived from the type of virus and the first letters of ID; the abbreviations H1-, H2-, and S- represent HIV-1, HIV-2, and SIV, respectively. Bioinformatic analysis was then performed by analyzing the occurrence of each sequence in other genomes using the Basic Local Alignment Search Tool (BLAST), a tool which can identify regions of local similarity between sequences [33]. The sequences summarized in Figure 1 were compared with the complete and partial genome sequences of all known HIVs available in the NCBI Gene database. Oligonucleotides numbers 1, 2, 4, 5, 6, 9, and 10 at the 3′-termini contain an additional proximal G_3_-run, and our criteria allow this to be included in the final sequence. However, the sequences marked by rectangles in such cases can represent the supposed loops of “standard” G-quadruplexes consisting of four G_*n*_-runs (*n* ≥ 3). However, the sequences of oligonucleotides used in experimental measurements are too short for relevant sequence alignment, as this approach is primarily used to examine sequences located in long terminal repeat regions (LTRs). Therefore, the wider regions were also used for bioinformatics analyses by adjusting the frame which is restricted by the NF-*κ*-B and TATA boxes for sequences located in LTRs. A similar methodology was also used for the translation products of the sequences located in coding regions.

### 2.2. Circular Dichroism Spectroscopy

CD spectra were recorded on a Jasco J-810 spectropolarimeter equipped with a PTC-423L temperature controller using a quartz cell of 1 mm optical path length in a reaction volume of 150 *μ*l. All other parameters and conditions were the same as those which were described previously [14, 33]. The modified Britton-Robinson buffer (mBR) was used in all spectral analyses where TRIS was used instead of potassium/sodium hydroxide (25 mM phosphoric acid, 25 mM boric acid, and 25 mM acetic acid) and supplemented by either 50 mM potassium chloride or sodium chloride. pH was adjusted by TRIS to a final value of 7.0.

DNA titration was performed with increasing concentrations of Thiazole Orange (TO). TO was solubilized in DMSO to reach a final concentration of stock solution of 10 mM. The concentrations of DNA and TO in a 1 mm quartz cell were 30 *μ*M and 0–200 *μ*M, respectively, and the increment of TO was ~67 *μ*M. Each sample was mixed vigorously for 3 min following the addition of TO; CD/UV spectra were measured immediately.

### 2.3. CD Melting Curves

CD melting profiles were collected at ~295 and ~265 nm as a function of temperature, using a procedure which has been published previously [33]. The temperature ranged from 0 to 100°C, and the heating rate was 0.25°C per minute. The melting temperature (*T*_*m*_) was defined as the temperature of the midtransition point.

### 2.4. Thermal Difference Spectra

The conditions and parameters used in the examination of the thermal difference spectra were identical to those used in the CD spectroscopy assay. The spectra analysis performed in this study has been described in an earlier publication [34].

### 2.5. Electrophoresis

Native polyacrylamide gel electrophoresis (PAGE) was performed in a temperature controlled vertical electrophoretic apparatus (Z375039-1EA, Sigma-Aldrich, San Francisco, CA). Gel concentration was 12% (19 : 1 monomer to bis ratio, Applichem, Darmstadt). Approximately two micrograms of DNA was loaded onto 14 × 16 × 0.1 cm gels. Prior to loading, each DNA sample was heated to 95°C for 5 min in an appropriate buffer and cooled to room temperature. Electrophoreses were performed at 20°C for 4 hours at 120 V (~8 V·cm^−1^). DNA oligomers were visualized with Stains-All immediately after electrophoresis, and the electrophoretic record was photographed on a white pad with a Nikon D3100 camera. The gel was also later stained by silver staining procedure in order to improve the sensitivity of the DNA visualization [15, 35].

## 3. Results and Discussion

### 3.1. Bioinformatic Analysis

**Figure 3:**
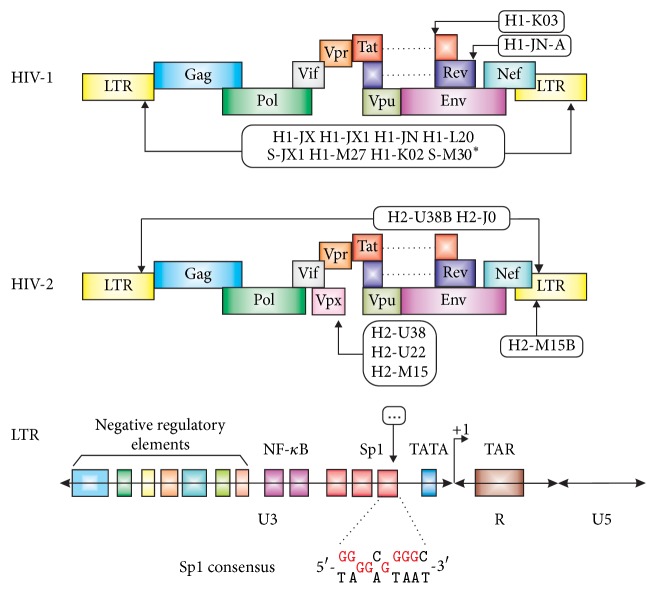
Schematic drawing of the HIV-1 and HIV-2 genome organizations and locations of studied sequence.

Although many different HIV sequence comparisons have been performed to date, this study offers an alternative means of identifying putative G-quadruplex forming sequences. The search criteria were not restricted to the LTR regions of the proviral HIV genome, but were instead applied to the entire proviral HIV genome. In this overview, the occurrence of 16 selected oligonucleotide sequences within more than nine thousand previously sequenced HIV/SIV genomes was examined in an attempt to identify some general relationship between them. The sequence structure consisting of three G-runs containing at least three neighboring Gs and one G-run containing two Gs was found in HIV-1, HIV-2, and SIV provirus DNAs. These sequences and their sources are summarized in Figure 1. Some sequences were also found in other organisms, but some of these are obligatory, located only in an appropriate HIV genome (Figure 3).

#### 3.1.1. The Vpx Region

The sequence H2-U22 was found only in one HIV-2 isolate in the terminal part of the vpx region (ID: U22047.1), but its derivatives containing 1–3 point mutations were found in an additional 9 HIV-2 isolates. H2-U38 is a truncated version of H2-U22, and this sequence was found again in the same region in an additional five HIV-2 genomic sequences (ID: M30502.1, U38293.1, M31113.1, U22047.1, and KU168289.1). The first 20 nucleotides of H2-M15 are identical to those of the H2-U22 and H2-U38 sequences, and this oligomeric sequence occurred very rarely in HIVs, being found in only 2 isolates of HIV-2 in vpx region (ID: X05291.1, M15390.1) and two derivatives containing 1-2 mutations. Interestingly, both sequence derivatives can also be found in other organisms. Considering the extreme rarity of these three sequences in HIV-2 genomes, their significance and biological role are questionable.

#### 3.1.2. Env/Rev Region

The G-rich region located in the env gene of HIV-1 is also a promising potential source of G-quadruplex formations. The env and rev coding sequences overlap, but their reading frames are different. This region contains the H1-JN-A sequence which occurs in only 11 HIV-1 isolates, but a derivative in which the central guanosine is substituted for adenosine (AGGGACTGAG**A**CTGGGGTGGGA) occurs in more than 1000 HIV-1 isolates. Interestingly, our results confirm that the formation of G-quadruplex is not affected and this sequence adopts preferentially the dimer form (not shown). There is some analogy here between the abasic site in the second G-run and the G for A substitution which has been the subject of recent studies by two different groups [36, 37]. The abasic site and the G for A substitution decrease the thermodynamic stability of such derivative sequences. Therefore, this substitution might not be sufficient to prevent such sequences from forming G-quadruplex motifs. It should be possible to verify the influence of a G for A substitution in the second G-run on a series of oligonucleotides. These results lead us to form the hypothesis that the formation of G-quadruplexes, as with the formation of other secondary motifs, could lead to a pausing effect on the DNA replication, transcription, or translation of the env and rev regions [38–40].

A number of different research projects have attempted to identify conserved structural motifs in highly variable viruses which can be used as specific targets for the development of efficient antiviral therapies. Interestingly, H1-JN-A sequence encodes the oligopeptides Gly–Leu–Arg–Leu–Gly–Trp–Glu and Gly–Thr–Glu–Ala/Thr–Gly–Val–Gly which are integral parts of Env and Rev proteins, respectively. These oligopeptide motifs are highly abundant in HIV-1 proteins and were found in more than 1140 coding sequences of Env and Rev.

#### 3.1.3. The Minus-Strand

All of the sequences described thus far are found in the plus-DNA/RNA strand. However, the sequence 5′-ACCCACCTCCCAACCCCG-3′ is typically located in the plus-strand of HIV-1 at the beginning of the second exon of tat/rev and env genes. This motif is complementary to the sequence 5′-d(CGGGGTTGGGAGGTGGGT)-3′ in the minus-strand and, interestingly, is very similar to the well-known HIV-93del aptamer d(GGGGTGGGAGGAGGGT), which forms very stable interlocked dimeric G-quadruplex [41]; the two sequences differ in two bases. Additionally, 98 known HIV-1 isolates differ only in one extra thymine compound between the first two G-runs, for example, isolate KU168259.1 in Gene bank. The H1-K03 sequence is located in 1160 various HIV-1 isolates.

Although the homology of H1-K03 and aptamer sequences is undoubtedly interesting, we are unable to offer a convincing explanation for the phenomenon. Nevertheless, this is the first reported case of a natural coding sequence being homological to an aptamer which was originally developed against the protein produced by the same organism. Is this merely a coincidence or is it an exception? However, if the sequence was located in the coding strand, it would be possible to elucidate an explanation or a convincing theory about the biological role of the sequence.

#### 3.1.4. LTR Regions

In recent years, a wide range of research and publications has focused on the study of G-rich sequences in LTR. In principle, the results of our research into U3 LTR sequences fully corroborate the earlier findings of other authors [2–4, 6, 7]. The H1-L20, H1-JX, and H1-JX1 sequences are very similar; indeed, H1-JX and H1-JX1 differ only in one central nucleobase. These sequences were found in HIV-1 genomes in LTR Sp1 region. Their occurrences are 8 and 9 hits for H1-JX and H1-JX1, respectively. Their derivatives were found in more than 30 various HIV-1 isolates. H1-L20 was found in only 5 isolates, although variants containing 1-2 point mutations were located in an additional 28 isolates. The formula implying all possible variants of HIV isolates which overlay H1-L20, H1-JX, and H1-JX1 sequences is

(1) $$\begin{array}{c} 5^{\prime }\text{-}TGGGAGGRAYRDKGGGYGGDHSDGGGA\text{-}3^{\prime }, \end{array}$$

where the following nomenclature of wobbles in DNA nucleobases is used: R = (A or G), B = (C or G or T), Y = (C or T), D = (A or G or T), S = (G or C), H = (A or C or T), W = (A or T), V = (A or C or G), K = (G or T), M = (A or C), and N can be any base.

The H1-JN sequence was found in a significantly higher number of HIV-1 variants, 3181 hits in HIV-1 genomic sequences and only one in the SIV isolate. This sequence was not found to occur in HIV-2 and other organisms. Recent studies have identified and described the structure of HIV-1 sequence LTR-IV: d(CTGGGCGGGACTGGGGAGTGGT) and their derivatives [42], and the underlined nucleotides of this sequence are homological to H1-JN sequence. The parallel G-quadruplex containing the bulge was confirmed by NMR analysis (PDB ID: 2N4Y).

H1-K02 and H1-M27 sequences are highly homological. These sequences partially overlap with H1-JN; their occurrence in HIV-1 genome was again found to be very high and is identified in more than 1700 various isolates in the NCBI database. The first guanosine is highly conservative in both sequences. This guanosine could be essential for the formation of G-tetrads and may also contribute to the stability of G-quadruplexes exhibiting bulge features. The large size of the statistical set would suggest the likelihood of higher numbers of nucleobase variations, and this was confirmed by the BLAST analysis of the region occurring between NF-*κ*-B and TATA boxes of KJ849802.1, in which H1-JN was found to occur in 1061 various isolates. All possible variants express the formula

(2) $$\begin{array}{c} 5^{\prime }\text{-}N\text{-}RDDVNHDVGSHGRRRHNNRRGADKSVB\text{-}3^{\prime }. \end{array}$$

The number of sequenced HIV-2 genomes was markedly smaller in comparison to that of HIV-1 and this inevitably resulted in a smaller statistical set. The sequence H2-U38B is a truncated version of H2-M15B; with the exception of the terminal 5′-adenosine of H2-J0, the two sequences are a perfect overlap. Their occurrence in different isolates was not as frequent as H1-JN, possibly due to the smaller number of sequenced HIV-2 genomes; all the identified sequences occurred in the LTR of HIV-2 and not in HIV-1. The truncated H2-U38 and H2-J0 derivatives were not only limited to humans, but also found in many other organisms such as* Ovis canadensis*,* Macaca fascicularis*,* Mus musculus*,* Rattus norvegicus*,* Mesocricetus auratus*, and* Fundulus heteroclitus* and pigs and plants such as* Solanum lycopersicum* and* Oryza punctate*. 145 different isolates of HIV-2 were found in the wider region determined with the NF-*κ*-B and TATA boxes. The formula including all possible variants is as follows:

(3) $$\begin{array}{c} 5^{\prime }\text{-}DRRGWRRNRYYTRGRRRGDRYYRGKRRGGA\text{-}3^{\prime }. \end{array}$$

The total number of all known HIV-1 sequences is much higher than the number of sequenced SIV genomes, but although the S-JX sequence originally found in SIV occurred prevalently in HIV-1 isolates, this was not the case for S-M30 sequence. Both S-JX and S-M30 sequences were also located in the LTR Sp1 region. S-M30 was found only in one SIV isolate, but its derivatives were found in an additional 6 SIV genomes and in other organisms including* Pseudomonas*, elephant endotheliotropic herpesvirus,* Enterobacter cloacae*,* Streptomyces leeuwenhoekii*, and* Leptosphaeria maculans* lepidii.

**Figure 4:**
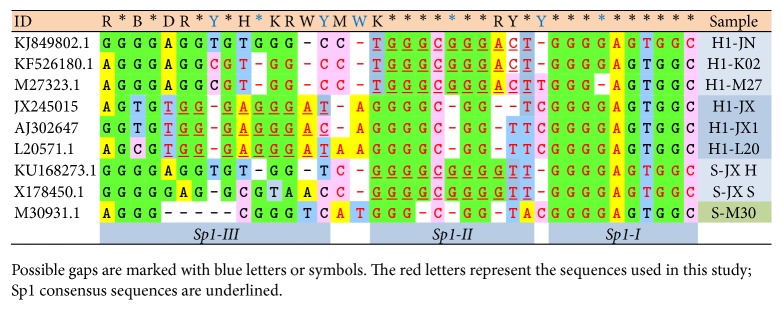
Sequence alignment of LTR regions containing the sequence listed in Figure 1. Possible gaps are marked with blue letters or symbols. The red letters represent the sequences used in this study. Sp1 consensus sequences are underlined.

The sequence alignment of the region containing the sequences used in this study, located in LTR of HIV-1 and SIV, is summarized in Figure 4. The alignments of the wider region determined by NF-*κ*-B and TATA boxes are shown in the Supporting Information (Table S1).

The formula including all possible variants is as follows:

(4) $$\begin{array}{c} 5^{\prime }\text{-}NDRGGRHGGGRRYYDRGGAGTGG\text{-}3^{\prime }. \end{array}$$

The models describing the formation of four G-quadruplexes formed in the LTR region have been described in a recent study [1, 6, 7]. The structure of one of these, an antiparallel G-quadruplex structure composed of only two tetrads, was also confirmed using NMR [4]. These four topologies are mutually exclusive because the G-runs associated with the formation of a single G-quadruplex are overlaid with others, thereby preventing the formation of any of the three alternative conformations. This sequence of four G-runs or more arranged in tandem is not uncommon in viral genomes, and similar regions consisting of 5-7 G-runs have also been found in human papillomaviruses [15]. Thus, the equilibrium between these forms may play a role in regulating promoter activity in viruses.

Targeting G-quadruplexes including the possible variations located in the LTR coding sequence of HIVs can therefore offer an attractive therapeutic opportunity for the development of highly efficient inhibitors of processes depending on the secondary motifs in this regulating region.

### 3.2. CD Measurements

**Figure 5:**
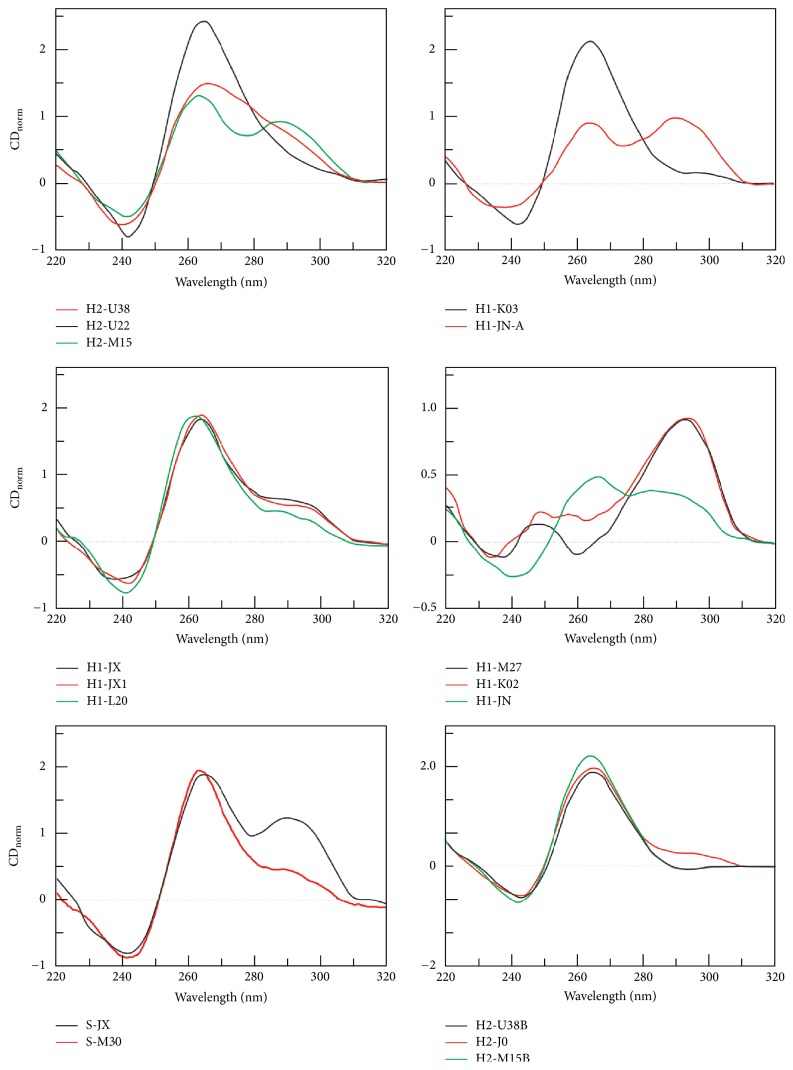
CD spectra of HIV oligomers in modified 25 mM mBR buffer (pH 7.0) in the presence of 50 mM KCl. The corresponding UV, TDS, and CD melting curves obtained at 265 and 293 nm are shown in Supporting Figures S1 and S2.

Another aim of this study was to confirm the ability of the studied oligonucleotides to form stable G-quadruplexes. In order to ascertain this, a series of experiments using circular dichroism analysis was performed (Figure 5). DNA oligonucleotides were analyzed in a buffer supplemented with 50 mM of potassium and sodium ions. All the studied oligonucleotides showed signatures which are typical for the formation of G-quadruplex structures in the presence of 50 mM KCl; positive CD peaks were recorded at ~265 nm and/or 295 nm. The comparison of relative molar intensity of CD peaks with human telomeric repeats sequence at 265 or 295 nm shows that the sequence which folds into G-quadruplex can consist of three tetrads [34]. All CD spectra and CD melting profiles obtained in the presence of sodium and potassium are summarized in Supporting Figure S1.

### 3.3. Electrophoretic Analysis

**Figure 6:**
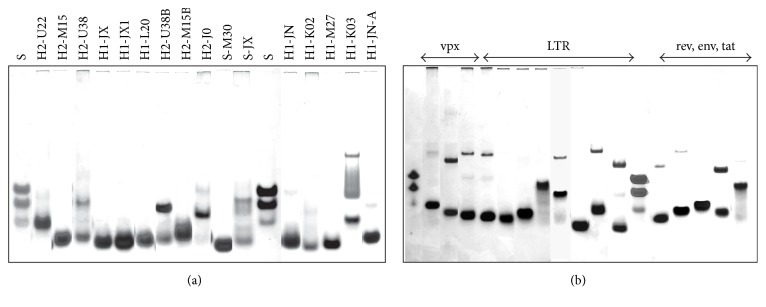
Molecular standard S, the mix of d(AC)_9_, d(AC)_14_, and d(AC)_18_, was used. Electrophoretic separation was performed in a 14% polyacrylamide gel at 10°C in 25 mM Britton-Robinson buffer (pH 7.0) and 50 mM KCl at 8°C in (a) and 50 mM NaCl in (b). Prior to being used, the DNA sample was heated in the same buffer for 5 min at ~98°C and slowly cooled to room temperature within 30 min.

Information about the molecularity and the presence of multimeric conformers of G-quadruplexes can be obtained by examining samples using electrophoretic separation [43]. Electrophoreses were performed in the presence of 50 mM KCl and NaCl at 20°C (Figure 6). DNA oligomers d(AC)_9_, d(AC)_14_, and d(AC)_18_ were used as molecular standards. Under identical conditions to those used in spectral measurements, most sequences were found to form exclusively intramolecular structures in the presence of potassium ions (Figure 6(a)). These forms move faster than the molecular standard representing the unfolded ssDNA. However, in the presence of sodium ions, smaller populations of dimeric forms are typically identified (Figure 6(b)). Dimeric structures in the presence of potassium were also observed for H2-U38, H2-U38B, H2-J0, and H1-K03. The results are summarized in Table 1.

Interestingly, the fastest band of H1-K03 represents a dimeric conformer, while the slowest and middle bands correspond to high-ordered structures. Preliminary NMR data indicates that H1-K03 forms an interlocked G-quadruplex structure which is analogical to HIV integrase aptamer [41]. The smearing of the middle band can be attributed to the presence of conformers with similar thermodynamic stabilities which possibly form one or more states during electrophoretic separation. The mobility of the dimer form of H2-M15B in the presence of sodium corresponds to the mobility of the unstructured molecular standards; therefore it is not possible to determine whether this form is a folded dimer or an unfolded dimer based on the electrophoretic record alone. However, the CD melting curve is clearly defined; this is possible only when the unknown structure transitions from one state to another; therefore it is possible to conclude that this band should represent a dimer structure (see Supporting Information, Figure S1).

It is important to note here, however, that not each band in a certain electrophoretic column necessarily represents a G-quadruplex structure.

### 3.4. TDS Analysis

The profiles of thermal difference spectra (TDS) of G-quadruplexes are highly specific, and therefore this analysis was also performed on the studied oligonucleotides, although, as our previous studies have noted, this technique is not wholly reliable and may provide erroneous results [34, 44]. The results are summarized in Supporting Figure S2 and in Table 1. The TDS results for H2-J0, H2-U38B, and H2-M15B were somewhat ambiguous but indicated the possible formation of G-quadruplexes in the presence of both sodium and potassium ions. Interestingly, their TDS show a maximum close to 260 mM in the presence of sodium, while the local minimum at 295 nm is not obvious in the presence of both sodium and potassium. Other oligonucleotides show a local minimum at 295 nm in TDS; for more information, see Supporting Figure S2.

More reliable results can, however, be obtained from melting curve analysis. The methodology of this technique is based on the fact that all known G-quadruplexes are more stable in the presence of potassium than in the presence of sodium ions [45]. The results of the melting curve analysis for our set of oligonucleotides revealed that the same sequences which offered interesting results in TDS assay, H2-J0, H2-U38B, and H2-M15B, also shared intriguing features in their melting curve profiles. Each of these sequences showed identical melting temperatures regardless of whether sodium or potassium ions were present in the used buffer. This raises the unanswered question of what type of secondary structure is formed within this set of oligonucleotides. A recent study described a novel tetrahelical structural motif which is distinct from the typical G-quadruplex structure, but this form has similar spectral properties as we observed in our experiments for these three oligonucleotides [46]. This study analyzed G_3_AGCG repeats found in the regulatory region of the PLEKHG3 gene and found that the VK sequences d(G_3_AGCGA)_*n*_G_3_AGCG, where *n* = 1,3, are capable of forming tetrahelical DNA stabilized with unusual noncanonical base-pairings: G-G and G-A. In addition, the sequences HPV25 and HPV25/2 found in the E4 gene of human papillomavirus type 25 also share many similar signatures [15]. These sequences prefer to adopt another fold as a G-quadruplex. Based on these findings, we decided to perform a sequence alignment of HPV25, HPV25/1, H2-M15B, H2-J0, and H2-38B to assess the homology of the three sequences. The results show a relative high homology among the sequences, which indicates that these three HIV-2 sequences (H2-J0, H2-U38B, and H2-M15B) could form structures very close to those of the HPV25 sequence (see Supporting Table S2). The melting temperatures of VK and HPV25 sequences were not dependent on the type of metal cation [15, 46]. Extrapolating from this, it is possible to suggest that the structure may form a hairpin-like structure containing unusual G-G and G-A base-pairs as was confirmed in VK. In principle, the tetrahelical VK structure is a special case of crossed hairpin structure. It has been shown that polyethylene glycol 200 (PEG200) stabilizes G-quadruplexes in the presence of potassium and destabilizes double helical motifs [45]. However, PEG-200 significantly increases the melting temperatures of the H2-M15B, H2-J0, H2-38B, VK, and HPV25 sequences in the presence of potassium; see Supporting Figure S5. In order to solve this Gordian knot and determine whether the H2-M15B and H2-38B sequences adopt G-quadruplex or other structural motifs, we decided to use another experimental method.

### 3.5. CD Titration Analysis

**Figure 7:**
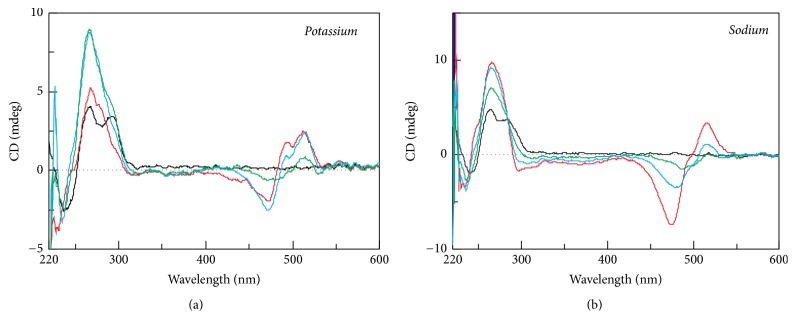
The representative CD titration spectra of ~27 *μ*M H1-JN with TO. 0, 2.5, 5, and 7.5 molar equivalents of TO represent black, green, blue, and red lines, respectively. Each sample was measured in modified 25 mM Britton-Robinson containing (a) 50 mM KCl and (b) 50 mM KCl.

Recently, a newly developed experimental methodology using the ligand Thiazole Orange (TO) for the identification of G-quadruplex forming sequences has been applied [47]. TO has a strong binding affinity to triplexes and G-quadruplexes, an affinity which is significantly higher to them than to other structural motifs [48, 49]. Although TO is optically inactive, TO-quadruplex complexes are chiral and display a unique profile of the induced CD (ICD) spectrum in the visible region [47]. This methodology offers valuable results in a wide range of conditions, but it is most sensitive in solutions without the presence of metal cations. Similarly, it can also be applied with slightly reduced sensitivity in solutions containing Na^+^ or low concentrations of K^+^ (<5 mM). Nevertheless, we performed the titration experiments in the presence of both 50 mM KCl and 50 mM NaCl because these concentrations of salts are more biologically relevant. The representative results of the titration analysis of H1-JN with TO in the presence of 50 mM NaCl and KCl are shown in Figure 7.

The ICD results display the expected positive signals at ~495 and ~510 nm and the negative signals at ~475 nm. These signatures are characteristic for TO-quadruplexes complexes [47]. The titration of all sequences is shown in Supporting Figures S3 and S4.

As expected, each oligonucleotide was also found to have formed G-quadruplexes under the given conditions. Signals corresponding to those of G-quadruplex structures were also clearly detected in the UV region. In case of antiparallel G-quadruplexes, the signals at 295 and 265 nm were seen to decrease and increase, respectively, by increasing the concentration of TO, phenomena which are indicative of the conversion from antiparallel to parallel folding.

**Figure 8:**
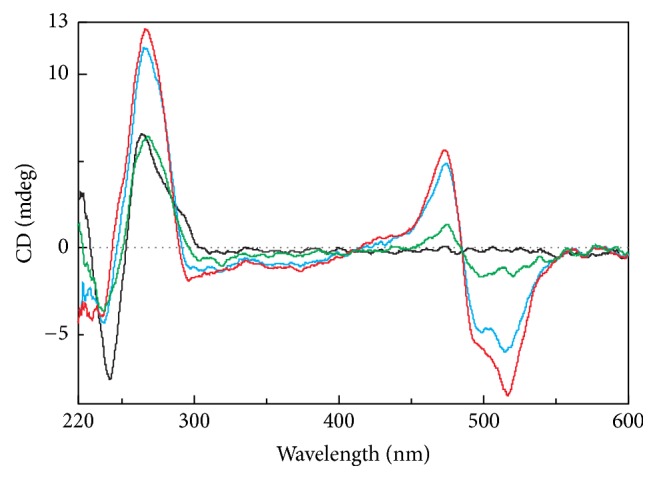
CD titration spectra of 27 *μ*M HPV25/1 (d(GGGAGCGGGAC-TGGGACCGGGACCG-GG)) with TO. 0, 2.5, 5, and 7.5 molar equivalents of TO are represented by black, green, brown, and red lines, respectively. Each sample was measured in a modified 25 mM mBR buffer containing 50 mM NaCl.

The titration analysis of the HPV25 sequence shows that ICD was mirrored; the positive peaks become negative and vice versa. It is therefore possible to assume that the binding mode of TO with this sequence must be different than those typical for G-quadruplex motifs (Figure 8). The same effect was also observed for VK sequence. These results indicate that H2-M15B, H2J0, and H2-U38B are also able to form G-quadruplex structures.

## 4. Conclusion

Our bioinformatic study of HIV genomes partially corresponds with the analyses recently published by many authors in that it too focuses primarily on G-rich regions located in U3 LTRs. However, this study reveals that G-quadruplexes can be formed in HIV provirus DNA when it only consists of three G-runs and one G_2_. These domains are not necessarily located only in regulating LTRs, but also in other gene coding regions. In addition, G-rich domains were also located in the minus-strand of many HIV-1 isolates, the sequence of which is highly homological with the well-known sequence forming the interlocked and extremely stable HIV integrase aptamer [41].

Several unanswered questions require deeper analysis to determine the features that provide specific G-quadruplex motifs with the ability to function as structural elements.

In this study, we used only the cost-effective methods to confirm that some oligonucleotides form G-quadruplex motifs. However, we again demonstrate that ICD signal of TO-quadruplex complex offers valuable additional information, allowing distinguishing whether an unknown sequence has ability to adopt G-quadruplex structure.

## Figures and Tables

**Table 1:** Melting temperatures and molecularities of the studied DNA oligonucleotides in the presence of 50 mM sodium and potassium ions.

		Potassium		Sodium	
	Oligo.	Fold	*T*_*m*_ [°C]	Fold	*T*_*m*_ [°C]
(1)	H2-U38	M, D	56.8^p^	M, D	52.4^p^
(2)	H2-U22	M	56.6^p^	M ≫ D	ND
(3)	H2-M15	M	57.1^a,p^	M, D	46.6^p^
(4)	H1-JX	M	57.5^a,p^	M > D	30.3^a^,52.5^p^
(5)	H1-JX1	M	61.6^a,p^	M > D	35.8^a^, 51.8^p^
(6)	H1-L20	M	58.2^p^	M	36.4^a^, 50.4^p^
(7)	H2-U38B	M < D	59.5^p^, 96.5^p^	M	55.3^p^
(8)	H2-J0	M, H ≪ D	51.8^p^, 87.0^p^	M, H ≪ D	50.8^p^
(9)	H2-M15B	M	46.5^p^	M^#&^ ≫ D	44.2^p^
(10)	S-M30	M	67.8^a,p^	M	49.6^a,p^
(11)	S-JX	M, D^#^	54.2^a^, 79.5^p^	M, D	35.0^a^, 39.2^p^
(12)	H1-JN	M	48.5^a^,46.5^p^	M > D	38.7^a^, 43.7^p^
(13)	H1-K02	M	48.4^a^	M > D	32.1^p^
(14)	H1-M27	M	39.6^a^	M	ND
(15)	H1-K03	D, H^#^	60.3^p^, <98^p^	M^&^, D	39.7^p^
(16)	H1-JN-A	M	58.6^a,p^	D ≫ M^#&^	37.2^p^

^#^Smear represents flexible conformers; ^&^the mobility can correspond to unfolded structure; M: monomer; D: dimer; H: high ordered multimer; ^p^melting curve was obtained at ~264 nm; ^a^melting curve was obtained at ~293 nm. The error in *T*_*m*_ determination is ±3°C.

## Supplementary Materials

Supplementary Materials Table S1: the sequence alignment of the regions determined by NF-*κ*-B and TATA boxes located in LTRs containing the sequences used in this study. Table S2: sequence alignments of HPV25, HPV25/1, H2-M15B, H2-J0, and H2-38B. Figure S1: the condition of CD spectra and melting curve measurements are the same as in Figure 3. Measurements in the presence of 50 mM NaCl and 50 mM KCl are represented as blue and black lines, respectively. The melting curves were obtained at 265 nm (solid lines) and 294 nm (dotted lines). The relative CD intensity is compared with the reference HTR oligonucleotide d(G3T2A)3G3. The value of 1 is obtained when the molar CD signal at 295 nm is the same as the CD signal of HTR at the same concentration [Tóthová et al. Biochemistry 53, 2014, 7013-27]. Figure S2: thermal difference spectra in the presence of sodium (blue lines) and potassium (black lines). Figure S3: the CD titration spectra of 27 *μ*M DNA sample with TO; 0, 2.5, 5, and 7.5 molar equivalents of TO are represented by black, green, blue, and red lines, respectively. Each sample was measured in a modified 25 mM mBR buffer containing 50 mM NaCl. Figure S4: CD titration spectra of 27 *μ*M DNA sample with TO. 0, 2.5, 5, and 7.5 molar equivalents of TO are represented by black, green, blue, and red lines, respectively. Each sample was measured in a modified 25 mM mBR buffer containing 50 mM KCl. Figure S5: CD spectra and melting curves of H2-M15B, VK, and HPV25/1 sequences in 50 mM KCl and the presence (magenta) and absence of 50% v/w PEG200 (black lines).
