# Supplementary material for: Putative HIV and SIV G-Quadruplex Sequences in Coding and Noncoding Regions Can Form G-Quadruplexes
Source: J Nucleic Acids. 2017 Dec 31;2017:6513720. doi: 10.1155/2017/6513720 (PMC5804116; doi:10.1155/2017/6513720)
Supplement: Supplementary Materials — Table S1: the sequence alignment of the regions determined by NF-κ-B and TATA boxes located in LTRs containing the sequences used in this study. Table S2: sequence alignments of HPV25, HPV25/1, H2-M15B, H2-J0, and H2-38B. Figure S1: the condition of CD spectra and melting curve measurements are the same as in Figure 3. Measurements in the presence of 50 mM NaCl and 50 mM KCl are represented as blue and black lines, respectively. The melting curves were obtained at 265 nm (solid lines) and 294 nm (dotted lines). The relative CD intensity is compared with the reference HTR oligonucleotide d(G3T2A)3G3. The value of 1 is obtained when the molar CD signal at 295 nm is the same as the CD signal of HTR at the same concentration [Tóthová et al. Biochemistry 53, 2014, 7013-27]. Figure S2: thermal difference spectra in the presence of sodium (blue lines) and potassium (black lines). Figure S3: the CD titration spectra of 27 μM DNA sample with TO; 0, 2.5, 5, and 7.5 molar equivalents of TO are represented by black, green, blue, and red lines, respectively. Each sample was measured in a modified 25 mM mBR buffer containing 50 mM NaCl. Figure S4: CD titration spectra of 27 μM DNA sample with TO. 0, 2.5, 5, and 7.5 molar equivalents of TO are represented by black, green, blue, and red lines, respectively. Each sample was measured in a modified 25 mM mBR buffer containing 50 mM KCl. Figure S5: CD spectra and melting curves of H2-M15B, VK, and HPV25/1 sequences in 50 mM KCl and the presence (magenta) and absence of 50% v/w PEG200 (black lines). [file 6513720.f1.docx]

**Supplementary Materials**

**Putative HIV and SIV G-Quadruplex Sequences in Coding and Non-Coding Regions can form G-quadruplexes**

*Petra Krafčíková, Erika Demkovičová, Andrea Halaganová and Viktor Víglaský**

Department of Biochemistry, Institute of Chemistry, Faculty of Sciences, P. J. Safarik University, 04001 Kosice, Slovakia

*To whom correspondence should be addressed.

E-mail: Phone: viktor.viglasky@upjs.sk

Phone: +421 55 2341262

Fax: +421 55 6222124

***Table S1:*** *The sequence alignment of the regions determined by NF-κ-B and TATA boxes located in LTRs containing the sequences used in this study.*

NF-κB Sp1 Sp2 Sp3 TATA ID

TGGGGACTTCCC◦◦◦◦GGGGAGGTGTGGCC**T**◦**GGGCGGGACTGGGGAGTGGC**GAG◦CCCTCAGATGCTGCATATAAG KJ849802.1 **H1-JN**

TGGGGACTTTCC◦◦◦◦AGGGAGGC**GTGGCCT**◦**GGGCGGGACTGGGGA**GTGGCGAG◦CCCTCAGATGCTGCATATAAG KF526180.1 **H1-K02**

TGGGGACTTTCC◦◦◦◦AGGGAGGC**GTGGCCT**◦**GGGCGGGACTTGGGA**GTGGCTAA◦CCCTCAGATGCTGCATATAAG M27323.1 **H1-M27**

CGGGGACTTTCCAGTG**TGGGAGG**◦**G**◦◦◦**ACAGGGGCGG**◦**TTCGGGGA**GTGGCTAA◦CCCTCAGATGCTGCATATAAG KU168283.1 **H1-JX1**

CGGGGACTTTCCAGTG**TGGGAGG**◦**G**◦◦◦**ATAGGGGCGG**◦◦**TCGGGGA**GTGGCTCA◦CCCTCAGATGCTGCATATAAG JX245015.1 **H1-JX**

CGGGGACTTTCCAGCG**TGGGAGG**◦**G**◦◦**ATAAGGGGCGG**◦**TTCGGGGA**GTGGCTAA◦CCCTCAGATGCTGCATATAAG L20571.1 **H1-L20**

CTGGGACTTTCC◦◦◦◦GGGGAGGTGTGGT**C◦GGGGCGGGGTTGGGGAGTGGC**TAA◦CCCTCAGATGCTGCATATAAG KU168273.1 **S-JX** (HIV1)

CGGGGA◦TTTCC◦◦◦GGGGGAGGCGTAAC**C◦GGGGCGGGGTTGGGGAGTGGCTAA**◦CCCTCAGATGCTGCATATAAG X178450.1 **S-JX** (SIV)

AAGGGACTTTCCAGGGCGGG◦◦◦◦◦◦◦**TCAT◦GGGCGG◦TACGGGGA**GTGGCTTTACCCTCAGA◦GCTGCATAAAAG M30931.1 **S-M30**

AAGGGACTTTCCAGGGCGGG◦◦◦◦◦◦◦CCAT GGGCGG◦TACGGGGAGTGGTTTTACCCTCAGA◦GCTGCATAAAAG M33718.1 **S-M30**

AGGGGCTGTAACCA◦◦**AGGGAGG◦GACATGGGAGGAGCTGGTGGGGA**AC◦◦◦◦◦◦GCCCTCATACTTACTGTATAAA [U38293.1](http://www.ncbi.nlm.nih.gov/nuccore/1845204) **H2-M15B**

The sequences of oligonucleotides used in our study are marked in red.

***Table S2:*** *Sequence alignments of HPV25, HPV25/1, H2-M15B, H2-J0 and H2-38B*

| Sequence | **** ***** ***** ** * * ** *** |
| --- | --- |
| **HPV25** | -**GGGA**GC**GGGAC**-**TGGGA**CC**GG**G**A**CC**G**-**GG**ACC**GGG** |
| **HPV25/1** | -**GGGA**GC**GGGAC**-**TGGGA**CC**GG**G**A**CC**G**-**GG** |
| **H2-M15B** | A**GGGA**--**GGGAC**A**TGGGA**--**GG**-**A**--**G**C**GG**T--**GGG**GA |
| **H2-U38B** | A**GGGA**--**GGGAC**A**TGGGA**--**GG**-**A**--**G** |
| **H2-J0** | G**GGGA**--**GGGAC**A**TGGGA**--**GG**-**A**--**G**C |

**
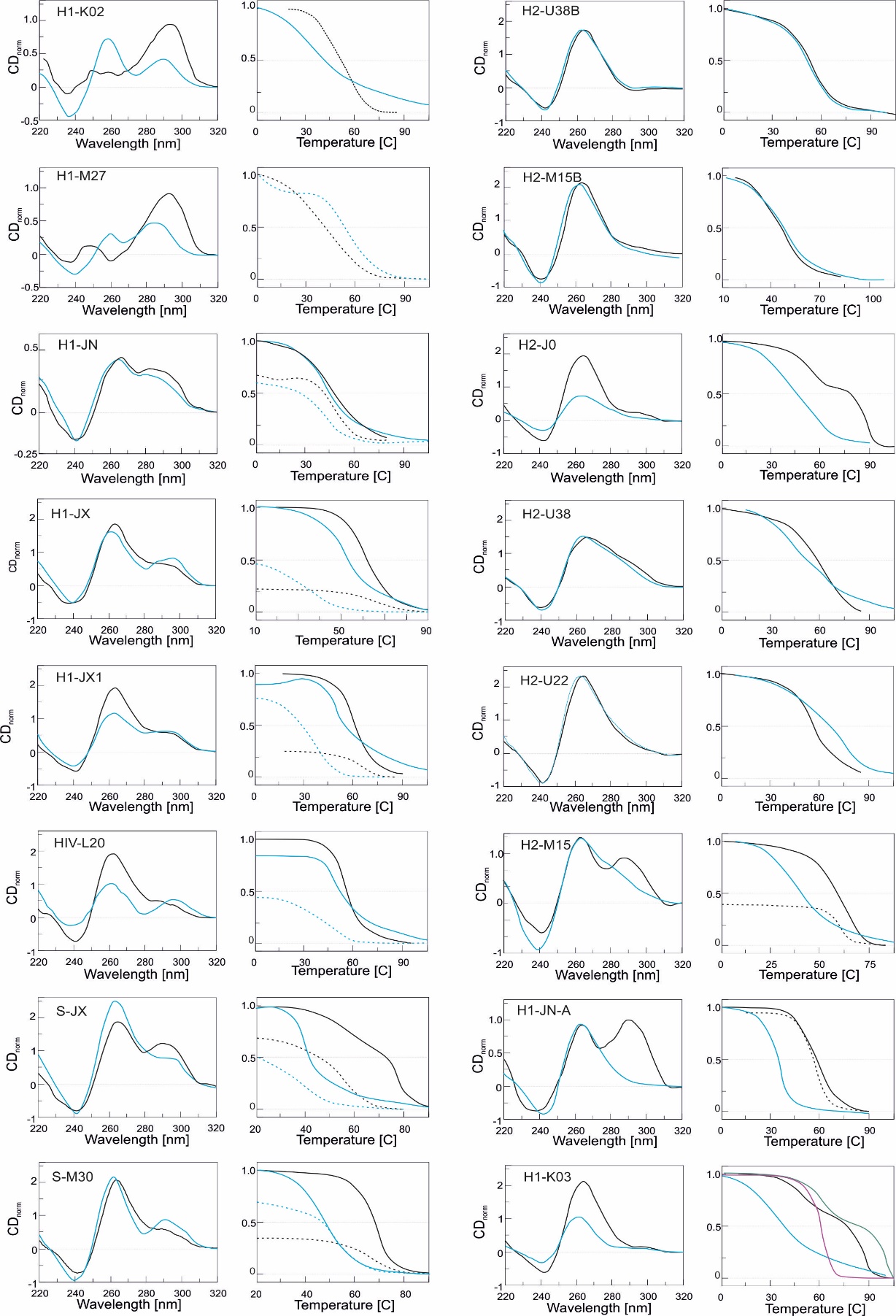
**

***Figure S1****. The condition of CD spectra and melting curve measurements are the same as in Figure 2. Measurements in the presence of 50 mM NaCl and 50 mM KCl are represented blue and black lines, respectively. The melting curves were obtained at 265 nm (solid lines) and 294 nm (dotted lines). The relative CD intensity is compared with the reference HTR oligonucleotide d(G3T2A)3G3. The value of 1 obtained when the molar CD signal at 295 nm is the same as the CD signal of HTR at the same concentration [Tothova et al. Biochemistry 53, 2014, 7013-27].*


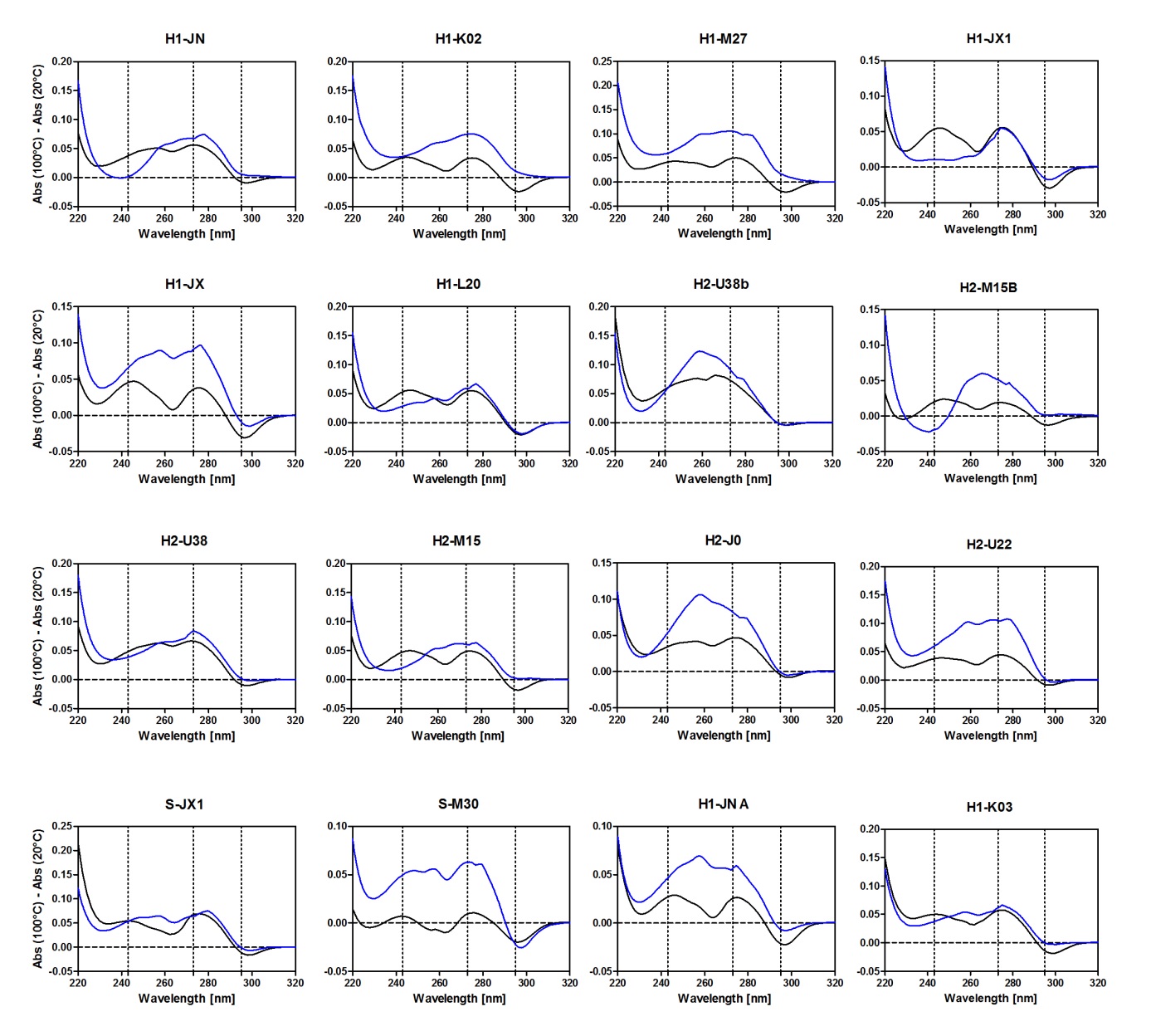


***Figure S2.*** *Thermal difference spectra in presence of sodium (blue lines) and potassium (black lines).*


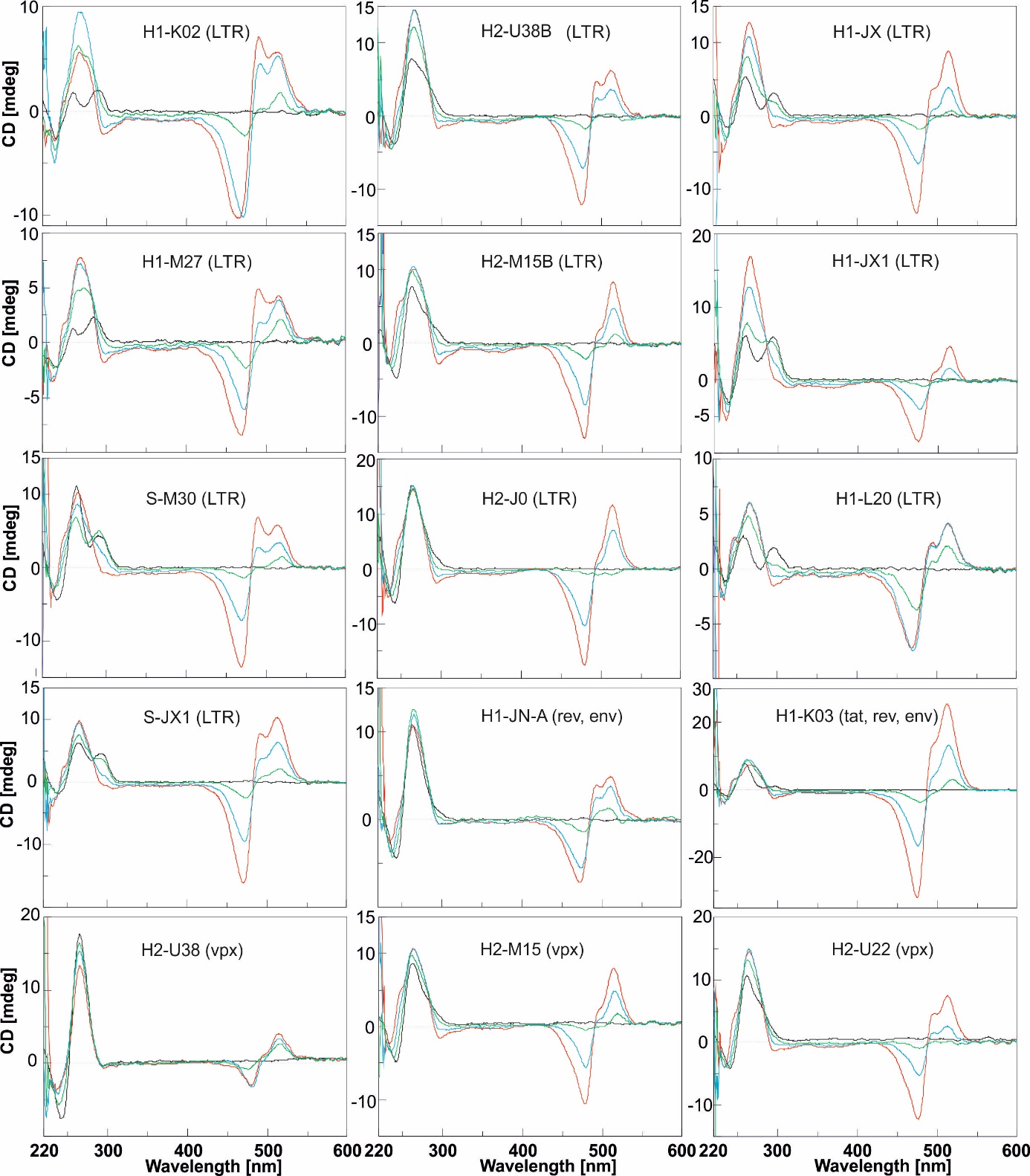


***Figure S3:*** *The CD titration spectra of 27 μM DNA sample with TO; 0, 2.5, 5 and 7.5 molar equivalents of TO are represent by black, green, blue and red lines, respectively. Each sample was measured in a modified 25 mM mBR buffer containing 50 mM NaCl.*


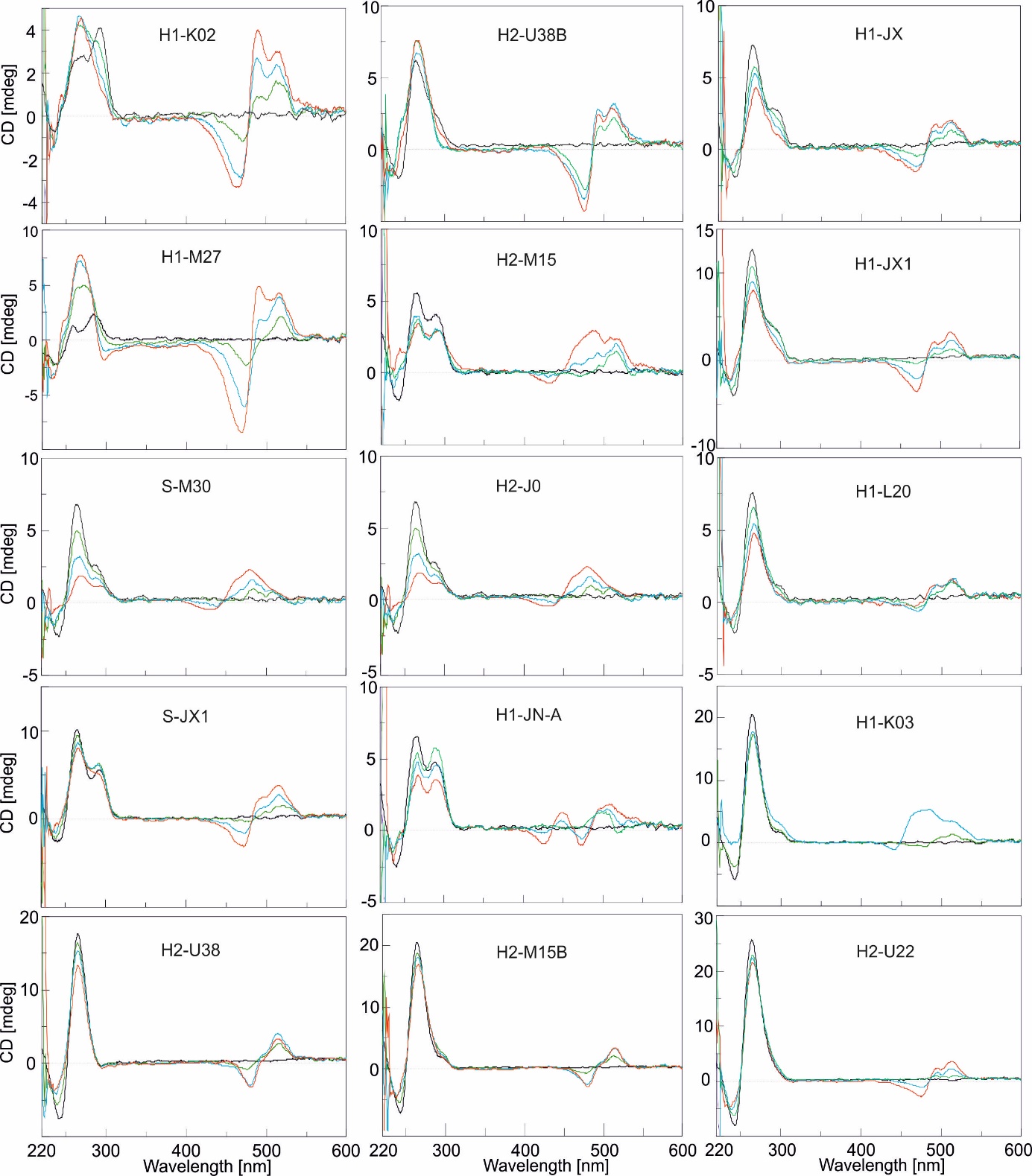


***Figure S4:*** *CD titration spectra of 27 μM DNA sample with TO. 0, 2.5, 5 and 7.5 molar equivalents of TO are represented by black, green, blue and red lines, respectively. Each sample was measured in a modified 25 mM mBR buffer containing 50 mM KCl.*


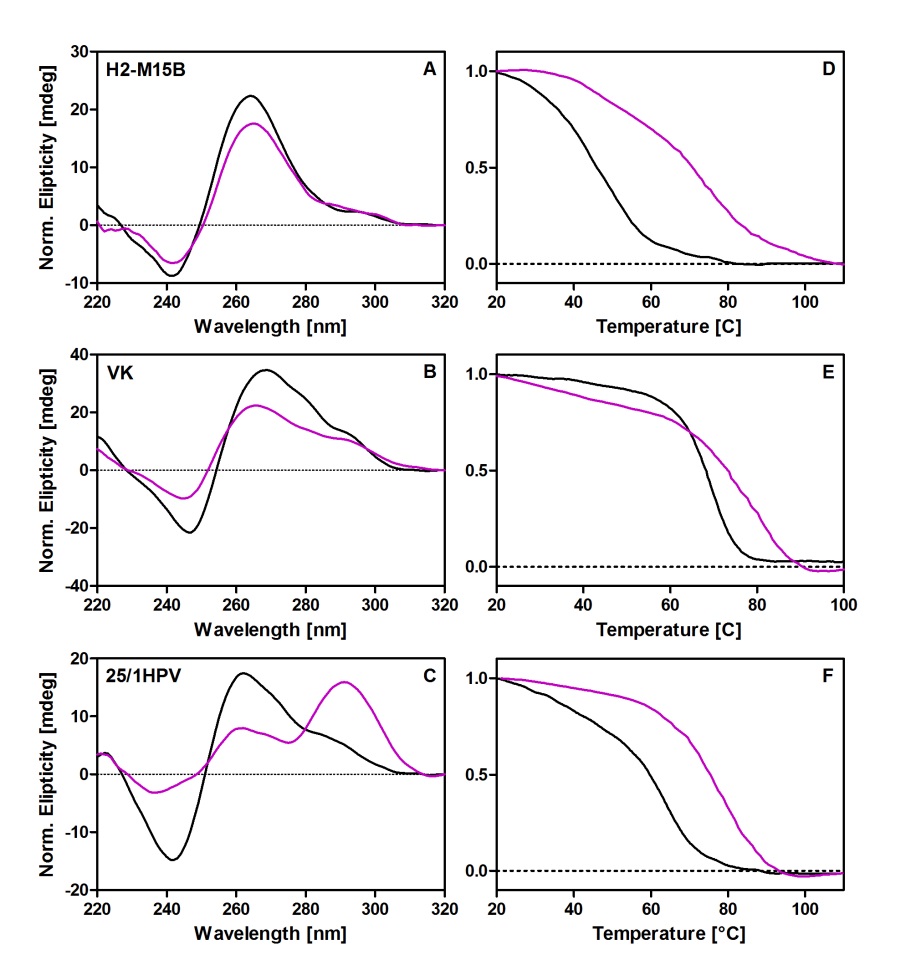


***Figure S5.*** *CD spectra and melting curves of H2-M15B, VK and HPV25/1 sequences in 50 mM KCl and presence (magenta) and absence of 50% v/w PEG200 (black lines).*
